# Ascending Reproductive Tract Infection in Pig-Tailed Macaques Inoculated with Mycoplasma genitalium

**DOI:** 10.1128/iai.00131-22

**Published:** 2022-05-18

**Authors:** Laarni Kendra T. Aguila, Dorothy L. Patton, German G. Gornalusse, Lucia N. Vojtech, Robert D. Murnane, Gwendolyn E. Wood

**Affiliations:** a Department of Medicine, Division of Allergy and Infectious Diseases, University of Washington, Seattle, Washington, USA; b Department of Obstetrics and Gynecology, University of Washington, Seattle, Washington, USA; c Washington National Primate Research Center, Department of Comparative Medicine, University of Washington, Seattle, Washington, USA; Yale University School of Medicine

**Keywords:** *Mycoplasma genitalium*, animal models, pelvic inflammatory disease

## Abstract

Mycoplasma genitalium is a sexually transmitted bacterial pathogen that causes urogenital disease in men and women. M. genitalium infections can persist for months to years and can ascend to the upper reproductive tract in women where it is associated with serious sequelae including pelvic inflammatory disease, tubal factor infertility, and preterm birth. An animal model is needed to understand immune evasion strategies that allow persistence, mechanisms of ascending infection, and factors associated with clearance. In earlier studies, we determined that pig-tailed macaques are susceptible to cervical infection; however, not all primates were successfully infected, persistence varied between animals, and ascension to the upper reproductive tract was not observed after 4 or 8 weeks of follow-up. Building on our previous findings, we refined our inoculation methods to increase infection rates, extended observation to 18 weeks, and comprehensively sampled the upper reproductive tract to detect ascending infection. With these improvements, we established infection in all (3/3) primates inoculated with M. genitalium and demonstrated lower tract persistence for 16 to 18 weeks. Ascension to the upper reproductive tract at endpoint was observed in two out of three primates. All three primates developed serum and local antibodies reacting primarily to the MgpB and MgpC adherence proteins. Elevated genital polymorphonuclear leukocytes (PMNs) and inflammatory cytokines and chemokines, erythema of the ectocervix in one primate, and histologic evidence of vaginitis and endocervicitis in two primates suggest a mild to moderate inflammatory response to infection. This model will be valuable to understand the natural history of M. genitalium infection including mechanisms of persistence, immune evasion, and ascension to the upper reproductive tract.

## INTRODUCTION

Mycoplasma genitalium is a bacterial pathogen first isolated from two men with nongonococcal urethritis (NGU) in 1980 (1). Since its isolation, M. genitalium has been recognized as a causative agent of NGU in men and associated with cervicitis and upper reproductive tract complications such as pelvic inflammatory disease (PID) in women (2). M. genitalium is increasingly resistant to first line therapies (3) and drugs used to treat PID have poor efficacy against M. genitalium (4). Significantly, studies have demonstrated a link between M. genitalium infection and HIV acquisition and transmission (5, 6), further highlighting the need to study this pathogenic bacterium and understand mechanisms of persistence and pathogenesis.

Typical M. genitalium infections are asymptomatic or present low-grade signs of inflammation, in fact, most patients are unaware of their infection (5, 7–9). A study that screened asymptomatic patients in a STI Health Clinic reported a positivity rate of 4.5% for M. genitalium, comparable to the positivity rate for Neisseria gonorrhoeae at 3.4% and Chlamydia trachomatis at 9.8% (7). The progression to the upper reproductive tract from unrecognized infection of the lower reproductive tract is an important reproductive health concern for women as upper tract complications such as PID are difficult to diagnosis. For instance, Taylor-Robinson et al. (10) demonstrated that clinical diagnosis did not correlate with laparoscopic findings in PID cases. In the case of M. genitalium infections, this appears to be further complicated by the 100-fold lower infectious load compared with C. trachomatis (11). Together, these studies highlight the need for improved criteria to identify and treat women at risk for upper reproductive tract morbidity due to an unrecognized M. genitalium infection.

M. genitalium persists for months to years in infected men and women despite the presence of M. genitalium-specific antibodies, suggesting that this pathogen evades the host immune response (12–14). Additional data from experimentally infected nonhuman primate models showed that humoral antibodies are insufficient to clear the infection (15, 16). The ability to evade the host immune response alongside the growing body of evidence associating M. genitalium with PID, emphasizes the clinical importance of this understudied bacterium. Despite the growing concerns, much of M. genitalium pathogenesis is still poorly understood.

Previous work by our group demonstrated that pig-tailed macaques are susceptible to M. genitalium lower reproductive tract infection. Of nine cervically inoculated primates, four were infected throughout the 8-week study, two were infected for 4 weeks, one for 1 week, and two resisted infection (16). M. genitalium-specific antibodies were produced by only a few animals. Furthermore, M. genitalium was not detected in upper reproductive tract biopsies collected after 4 and 8 weeks of infection in any of the animals. We hypothesized that optimizing the inoculum would improve the rate at which chronic M. genitalium infection was established and that extending the length of infection may be necessary to observe ascension to the upper reproductive tract. Here we report improvements to the pig-tailed macaque model that resulted in chronic lower tract infection and serum and local antibody responses in all three inoculated animals. In addition, ascension to the upper reproductive tract and histologic evidence of lower tract inflammation was observed in two of the three macaques.

## RESULTS

### Optimization of inoculum preparation methods.

**FIG 1:**
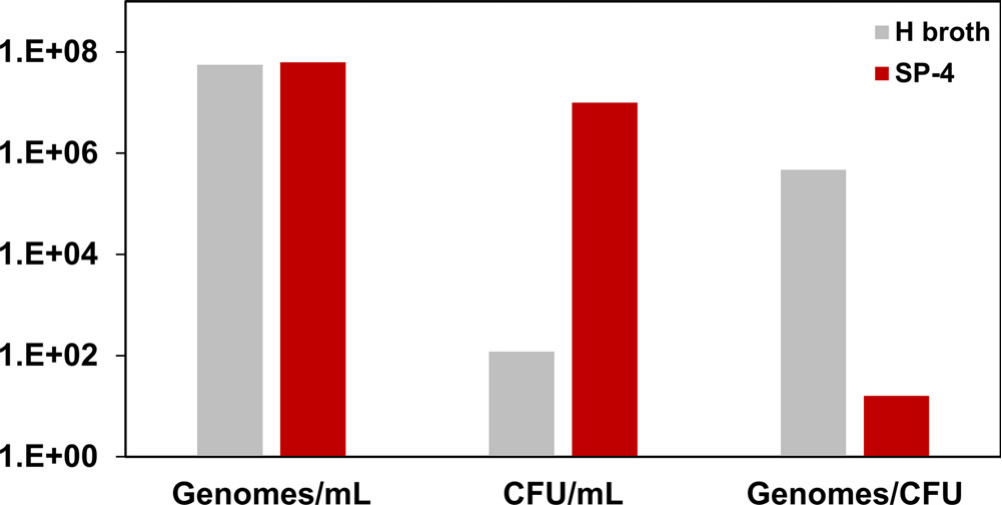
Growth medium affects aggregation of M. genitalium. Log phase cultures of M. genitalium grown in H broth (gray bars) or SP-4 broth (red bars) were compared by qPCR (genomes/mL) and plate counts (CFU per mL). Bars show the mean and standard deviation for a typical experiment (repeated three times).

To improve the infection rate of inoculated primates we considered the growth medium used to prepare the inoculum. We observed that M. genitalium cultured in H broth (used in previous studies [15, 16]) grows as large aggregates and adheres less to plastic than when grown in SP-4 broth. As shown in Fig. 1, M. genitalium grown in H broth and SP-4 reach similar genomes per mL but CFU per mL were 5-logs lower in H broth. The corresponding increase in genomes per CFU confirmed that, compared with SP-4 broth, H broth promotes aggregation of M. genitalium which may reduce colonization of the genital tract. Consequently, we used M. genitalium grown in SP-4 broth for inoculation of pig-tailed macaques.

### Lower genital tract infection of pig-tailed macaques.

The goals of these pilot experiments were to establish persistent cervical infection in female pig-tailed macaques and determine if M. genitalium can ascend to the upper reproductive tract. As M. genitalium is a fastidious, slow-growing organism, we used complementary strategies to track infection including detection of M. genitalium DNA by qPCR, direct culture of specimens in SP-4 broth, and coculture of M. genitalium with Vero cells. Analysis of pre-inoculation lower reproductive tract specimens confirmed that M. genitalium DNA and viable bacteria were absent in these primates. Primates were inoculated cervically with M. genitalium strain G37C (4-8 × 10^9^ genome equivalents) and then assessed weekly for 4 weeks and biweekly through week 18 for the presence of M. genitalium in vaginal, cervical, and endocervical specimens, and for induction of local and systemic immune responses in cervical secretions and serum.

As shown in Table 1, persistent lower genital tract infection was established in all three pig-tailed macaques. In primate Z13108, M. genitalium was detected by qPCR in at least one specimen at all time points except week 10. Primate Z15203 was PCR positive at all time points through week 16, and primate Z15014 was PCR positive at every time point through study endpoint (week 18). Overall, we detected M. genitalium DNA in 82/96 (85.4%) of the total lower reproductive tract specimens after inoculation.

**TABLE 1:** Recovery of M. genitalium from experimentally infected primates^*a*^

Primate no.		Z13108			Z15203			Z15014		
Week	Specimen^*b*^	SP-4^*c*^	Vero^*d*^	qPCR^*e*^	SP-4	Vero	qPCR	SP-4	Vero	qPCR
1	Vag	3	+	+	-	-	+	CM^*f*^	+	+
	Cx	>4^*g*^	+	+	3	-	+	>4	+	+
	CxCb	>4	+	+	>4	+	+	>4	+	+
2	Vag	>4	+	+	-	-	+	>4	+	+
	Cx	>4	+	+	>4	+	+	>4	+	+
	CxCb	>4	+	+	>4	+	+	>4	+	+
3	Vag	>4	+	+	-	-	+	>4	+	+
	Cx	>4	+	+	>4	+	+	>4	+	+
	CxCb	>4	+	+	>4	+	+	>4^*g*^	+	+
4	Vag	>4^*g*^	+	+	>4	+	+	>4	+	+
	Cx	>4	+	+	>4^*g*^	+	+	1	+	+
	CxCb	>4	+	+	>4^*g*^	+	+	>4^*g*^	+	+
6	Vag	>4^*g*^	+	+	-	-	+	>4^*g*^	+	+
	Cx	>4	+	+	2	+	-	>4^*g*^	+	+
	CxCb	>4	+	+	>4	+	-	>4^*g*^	+	+
8	Vag	1	-	-	>4^*g*^	+	+	>4	+	+
	Cx	-	-	+	1	-	-	>4	+	+
	CxCb	1	-	-	2^*g*^	-	-	>4^*g*^	+	+
10	Vag	-	-	-	2^*g*^	+	+	-	-	+
	Cx	-	-	-	3^*g*^	+	+	-	-	+
	CxCb	-	-	-	4^*g*^	+	+	-	-	+
12	Vag	-	-	+	-	-	+	3^*g*^	+	+
	Cx	-	-	+	-	-	+	4^*g*^	+	+
	CxCb	ND	ND	ND	-	-	+	1	-	+
14	Vag	-	-	+	-	-	+	3^*g*^	+	+
	Cx	-	-	+	-	-	+	3	+	+
	CxCb	-	-	+	-	-	+	ND	ND	ND
16	Vag	-	-	+	-	-	+	1	+	+
	Cx	-	-	+	-	-	+	-	-	+
	CxCb	-	-	+	ND	ND	ND	-	-	+
18	Vag	-	-	+	-	-	-	-	-	-
	Cx	2	-	+	-	-	-	-	-	-
	CxCb	-	-	+	-	-	-	-	-	+

a Primate numbers are indicated on the top. M. genitalium positive specimens are indicated as (+), negatives as (–). ND, not determined. Specimens collected prior to inoculation (weeks −3 and 0, not shown) were negative for M. genitalium by qPCR and culture.

b Specimen types: Vag, vaginal swab; Cx, cervical swabs; CxCb, endocervical cytobrush.

c Growth of M. genitalium in SP-4 was determined by color change in any of four 10-fold serial dilutions and microscopy to confirm M. genitalium colony morphology. Numbers indicate log number of color change units.

d Growth in Vero cell cultures, as determined by qPCR detection of M. genitalium genomes in 12 μL of culture supernatant.

e M. genitalium genomes detected in 12 μL of primate specimen as determined by quantitative PCR.

f CM, gross contamination.

g Growth of M. genitalium in SP-4 inhibited at lower dilutions of specimen.

We previously demonstrated that recovery of viable M. genitalium is enhanced by coculture with Vero cells compared with H broth (15, 16). In the current study, recovery of viable M. genitalium from the 96 lower reproductive tract specimens collected was similar with 57 (59.4%) and 52 (54.2%) positive in SP-4 broth and Vero cell cocultures, respectively (Table 1). In fact, M. genitalium grew in SP-4 broth but not in Vero cell cocultures for 7 out of 96 (7.3%) specimens. Positive M. genitalium cultures (SP-4 broth or Vero cocultures) were obtained from Z13108 through week 8 and again at week 18. Primate Z15203 was culture-positive through week 10. Positive cultures from primate Z15014 spanned 16 weeks, with the exception of week 10.

**FIG 2:**
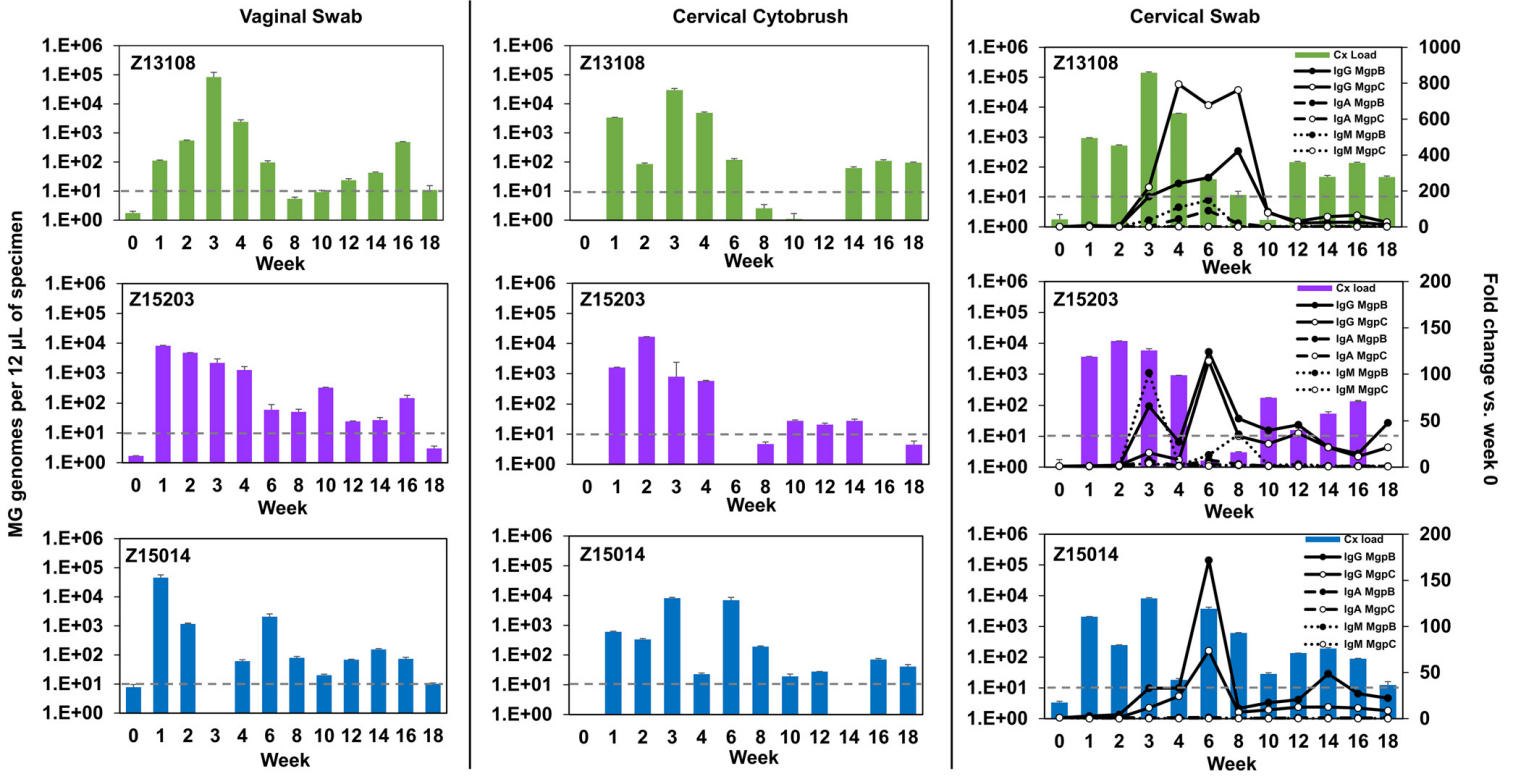
Longitudinal changes in M. genitalium organism load and antibody reactivity in primate lower reproductive tract specimens. Primate numbers and week postinoculation (bottom) are indicated. The *y* axis values indicate M. genitalium genomes detected by qPCR (bars) in vaginal swabs, endocervical cytobrush and cervical swab specimens. Specimens with more than 10 genome copies (gray dashed line) were considered M. genitalium positive based on comparison to pre-inoculation specimens. Lines in the right panel indicate fold change in IgG, IgA, or IgM reactivity to MgpB (closed circles) or MgpC (open circles) compared with week 0, as detected by immunoblot. IgG (solid lines), IgA (dashed lines), and IgM (dotted lines) immunoblot reactivity to MgpB and MgpC were quantified and relative measurements compared to week 0 are reported.

To quantitate viable bacteria recovered from the lower reproductive tract, four serial 10-fold dilutions of each specimen were cultured in SP-4 broth. Specimens from earlier time points (week <8) tended to grow in all four dilutions (Table 1), correlating with the high number of M. genitalium genomes detected (Fig. 2). As previously observed (16), growth was delayed in some cultures inoculated with lower dilutions (1:10 or 1:100) compared with higher dilutions (1:1,000 or 1:10,000), probably due to the presence of growth inhibitors in genital tract secretions (Table 1). This suggests that specimens with low numbers of viable M. genitalium would appear culture negative as the dilution necessary to eliminate inhibition would also reduce input bacteria. Consistent with this idea, of the 29 lower tract specimens that were PCR-positive but culture-negative, 26 had <200 genomes detected by qPCR. In contrast, vaginal swabs from Z15203 were culture negative at weeks 1, 2, and 3 despite high numbers of genomes and high titers of viable M. genitalium in cervical specimens at these time points. This may be explained by the lower vaginal pH (4.5 to 6.0) in Z15203 at weeks 1 to 3 which may have reduced the viability of M. genitalium in the vagina. In comparison, vaginal pH was higher in Z13108 (pH 7 to 7.5) and Z15014 (pH 6.5 to 8.5) which were culture positive in vaginal swabs at these time points.

### Detection of M. genitalium in the upper reproductive tract.

**FIG 3:**
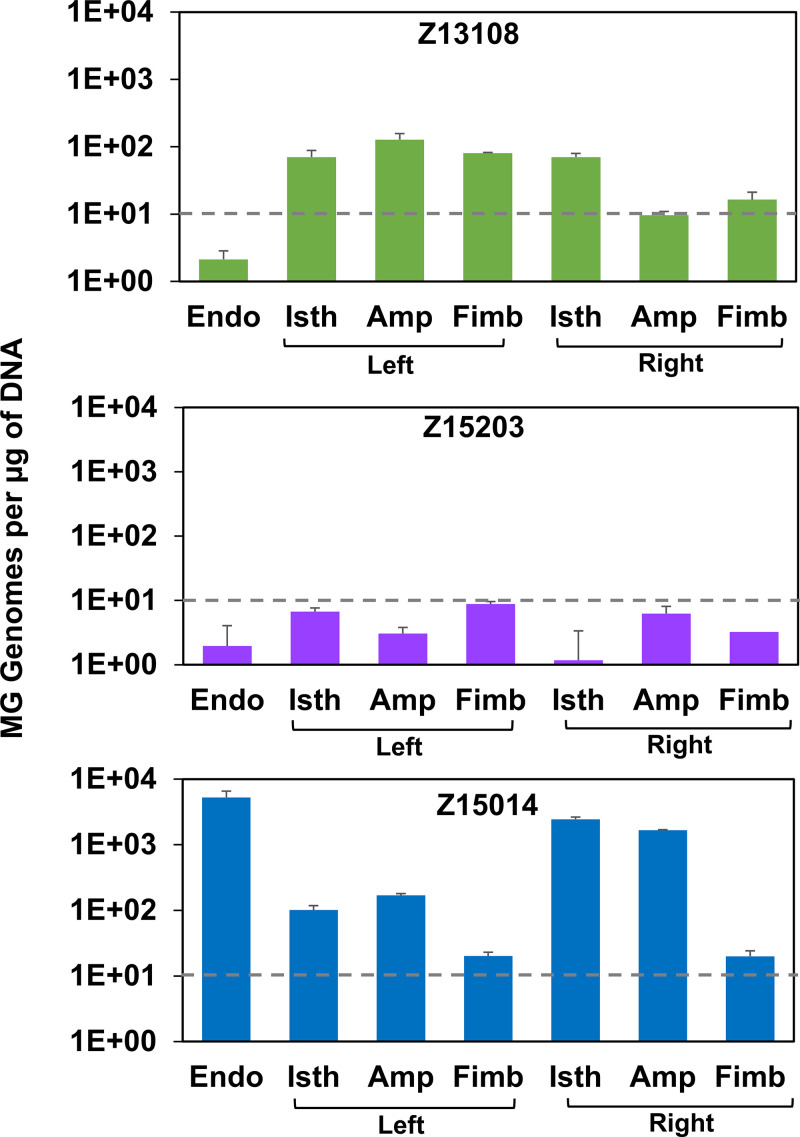
Detection of M. genitalium in upper reproductive tract tissues. The number of M. genitalium genomes recovered from upper reproductive tract tissues per μg of total DNA (to standardize between tissue fragments) is shown. Primate numbers are indicated on top and sample site are on the bottom of each graph. Values above the threshold (10 copies, gray dashed line) were considered M. genitalium positive. Endo, endometrium; Isth, isthmus; Amp, ampulla; Fimb, fimbriae.

Necropsy was performed 18 weeks after inoculation and comprehensive reproductive tract specimens were collected to detect ascending M. genitalium infection. Tissue samples were obtained from the endometrium and left and right fallopian tubes at three locations (isthmus, ampulla, fimbriae) then homogenized and analyzed for the presence of M. genitalium by qPCR. As shown in Fig. 3, M. genitalium was not detected by qPCR in any upper reproductive tract tissue from primate Z15203, consistent with the PCR-negative status of the lower genital tract specimens at week 18. In contrast, M. genitalium DNA (2.7 × 10^3^ to 2.1 × 10^4^ genomes per tissue fragment) was detected in all three regions (isthmus, ampulla, and fimbriae) of the left fallopian tube, and in the isthmus of the right fallopian tube in primate Z13108, but not in the endometrium. M. genitalium genomes were detected at even higher numbers (3.3 × 10^3^ to 8.8 × 10^5^ genomes per tissue fragment) in the upper reproductive tract of primate Z15014 including the endometrium and multiple regions of the left and right Fallopian tubes. No M. genitalium positive cultures were obtained from homogenized tissue suggesting either that growth inhibitors prevent growth *in vitro* or that PCRs detected DNA from dead bacteria. These results demonstrate for the first time that M. genitalium can ascend to the reproductive tract after cervical inoculation of pig-tailed macaques.

### Analysis of genital tract inflammatory response.

**FIG 4:**
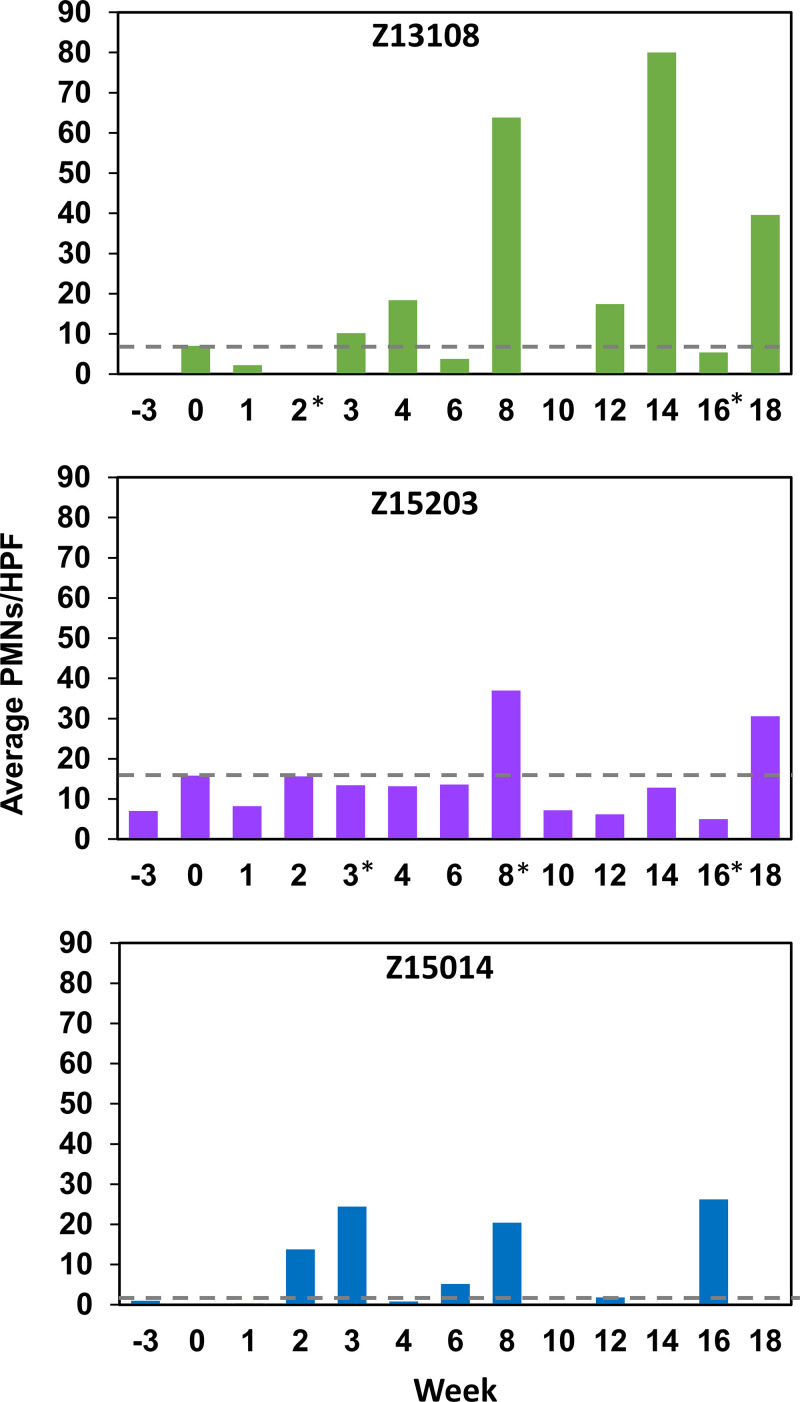
Average polymorphonuclear cells (PMNs) in five nonadjacent high-power fields (HPF, 400x) counted from endocervical cytobrush smears over the course of the study. Primate numbers are indicated on the top of each graph and week postinoculation (week 0) on the bottom. Baseline PMNs (gray dashed line) characteristic of each primate represent the highest PMN count in pre-inoculation (week −3 or 0) specimens. *, specimens containing blood.

The cervix of infected animals was examined by colposcopy for signs of inflammation at each time point. Erythema was observed at week 16 in Z15203, and bleeding was easily induced when collecting vaginal swab specimens from primate Z15014 at weeks 6, 12, and 16 suggesting the integrity of the vaginal epithelium was diminished. Abnormal cervical discharge was not observed at any time point. Cervical swab specimens were examined by differential staining and light microscopy to determine the number of polymorphonuclear leukocytes (PMNs) per high power field (HPF). Pre-inoculation samples (weeks −3 and 0) established baseline PMN counts, which varied between primates (Fig. 4). Primates Z13108 and Z15014 had low baseline PMNs averaging 0 to 7 and 0 to 1 PMN/HPF, respectively, whereas baseline PMNs in primate Z15203 were somewhat higher (7 to 17 PMNs/HPF). After inoculation, PMNs increased above baseline at five time points for Z13108 and Z15014, and at two time points for Z15203 (Fig. 4). These time points did not correspond to menses, suggesting intermittent induction of a cellular inflammatory response due to M. genitalium infection.

**FIG 5:**
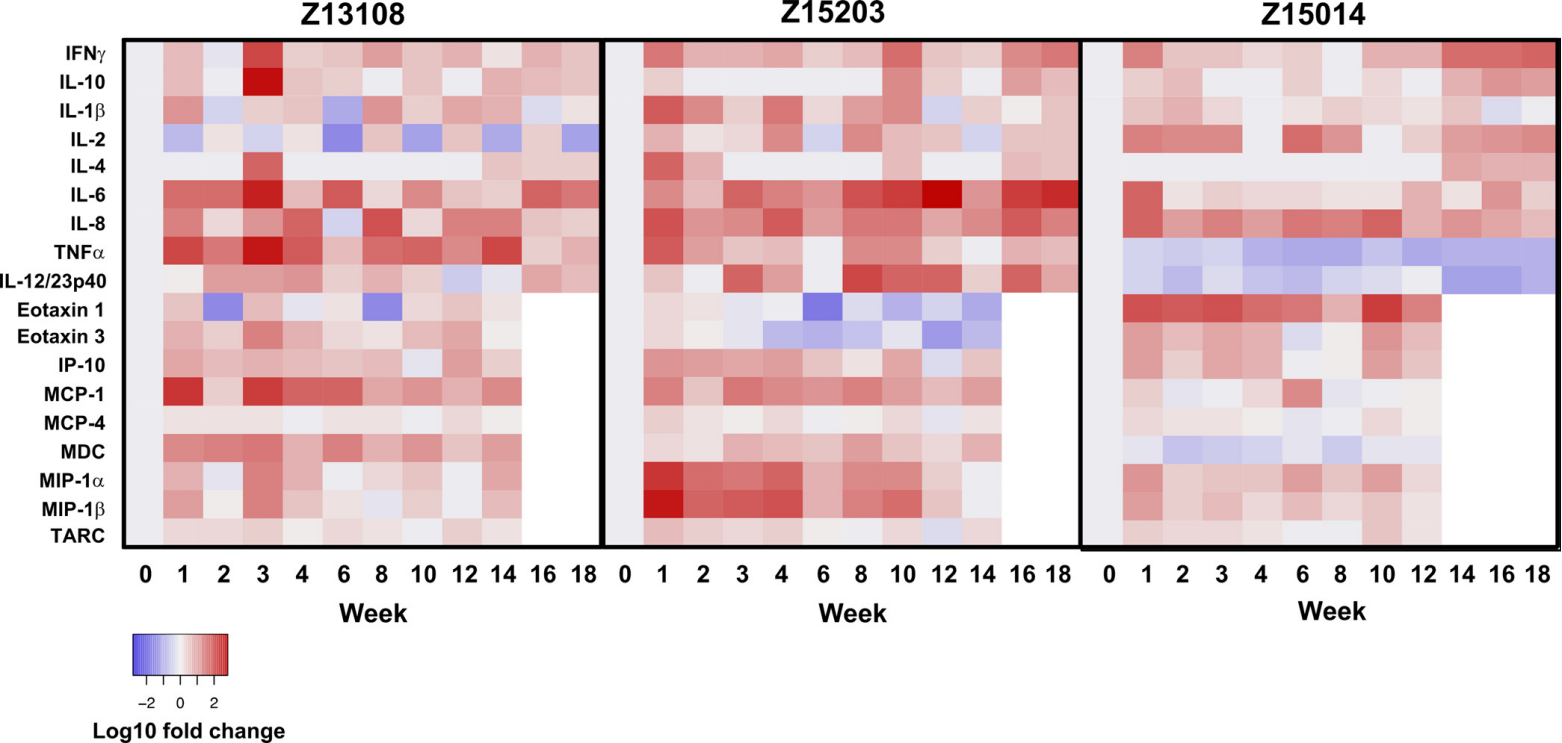
Heat map of inflammatory cytokine and chemokine expression from endocervical secretions collected over time. Primate numbers (top) and week postinoculation (bottom) are indicated. Changes in cytokine and chemokine expression are reported as log_10_ fold change compared to week 0 (gray). Chemokines at later time points were not measured (white).

Cytokines in endocervical secretions were quantified and compared to baseline (week 0) as shown in Fig. 5 and Fig. S1. The levels of many cytokines varied widely between individual animals and between time points. IL-8 was increased in all three primates: 1.8- to 67-fold in Z13108 (except an ~2-fold decrease at week 6), 3.8- to 45-fold in Z15203, and 4.2- to 43-fold in Z15014. In addition, primates Z13108 and Z15203 had early and persistent increases of IL-6, TNF-α, and MCP-1. IFN-γ, IL-1β, IL-6, TNF-α, MIP-1α, and MIP-1β increased in Z15203 with further induction of IL-6 observed at weeks 12 to 18. Fewer cytokines were increased in Z15014 with IFN-γ, IL-2, and Eotaxin-1 responses predominating.

**FIG 6:**
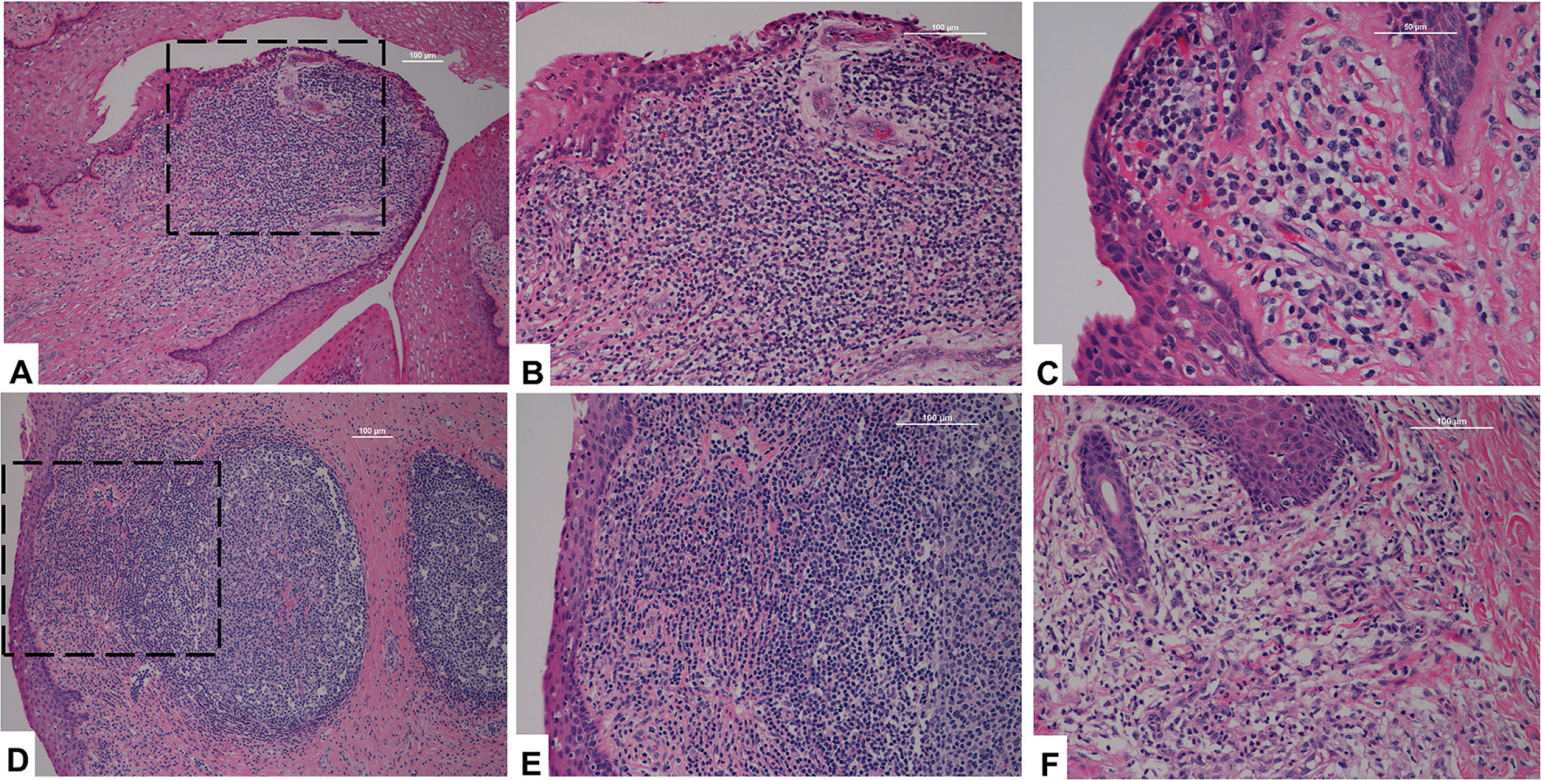
Histology of lower genital tract. Primate Z13108 (A to C) ectocervicitis with infiltration of lymphocytes and macrophages, and exocytosis. The boxed area in (A) is magnified 10x in (B). Primate Z15014 (D, E); submucosal infiltrates of lymphocytes and macrophages, and lymphofollicular hyperplasia. The boxed area in (D) is magnified 10x in (E). (F) shows labitis dermatitis.

Histologic evaluation of reproductive tract tissues obtained at endpoint (Fig. 6) revealed evidence of genital tract inflammation in two of the three animals. In Z13108, the ectocervix and distal vagina had mild to moderate submucosal and perivascular aggregates of lymphocytes and macrophages with epithelial infiltration (exocytosis) multifocally (Fig. 6A to C). Similar evidence of inflammation was observed in tissues of primate Z15014 (Fig. 6D to F): the distal vagina had mild, multifocal round cell infiltrates (with exocytosis). The mid to proximal vagina and labia had mild to moderate, multifocal, submucosal infiltrates of lymphocytes and macrophages with few plasma cells, and with exocytosis multifocally. We also found moderate to moderately extensive lymphofollicular hyperplasia in the proximal vagina and labia (Fig. 6D and E). Distal labia and skin had mild multifocal lymphohistiocytic submucosal and dermal aggregates (Fig. 6F), and in the dermis there was minimal suppuration as well. The lower and upper tract tissues of primate Z15203 appeared normal.

### Antibody response in M. genitalium infected primates.

**FIG 7:**
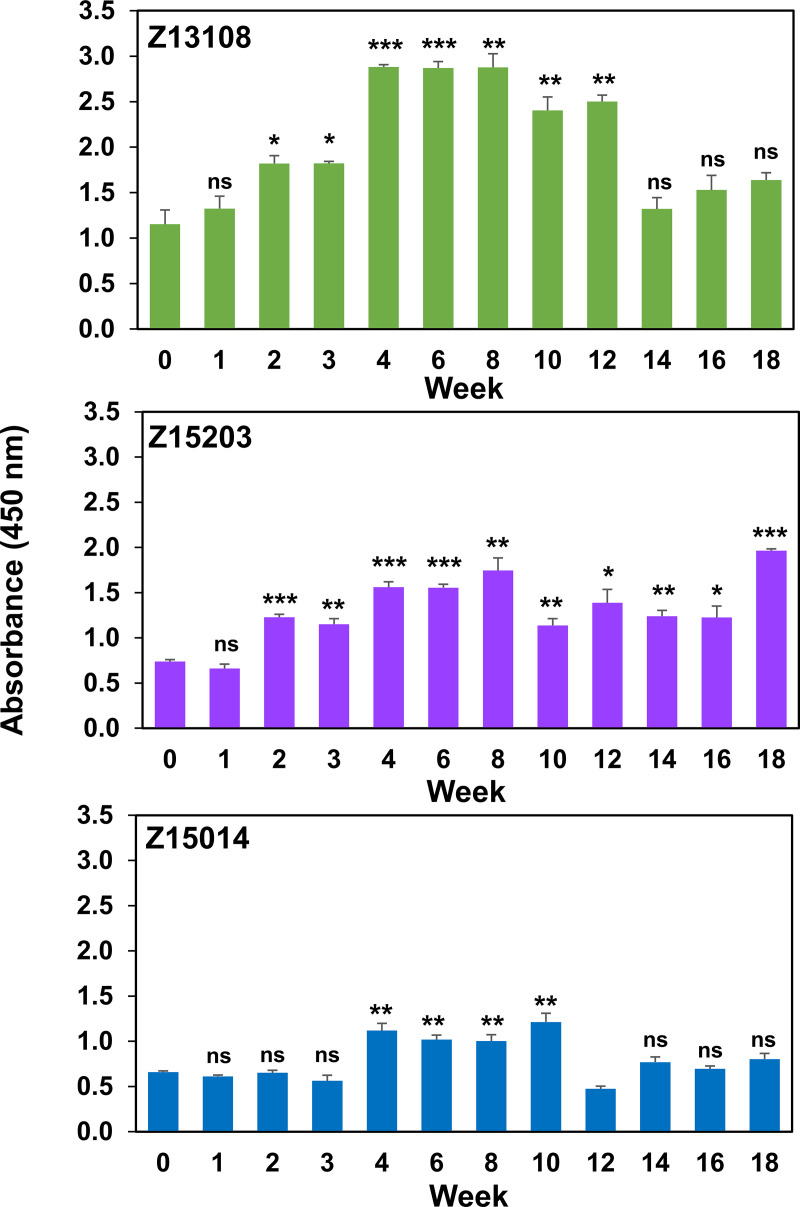
ELISA reactivity of primate sera against M. genitalium whole cells. Values shown are mean and standard deviation for triplicate measurements. Results of a typical experiment (performed three times) are shown. *P* values were calculated compared with week 0 using a two-tailed *t* test; ns, not significant (*P* > 0.05); *, *P* < 0.05; **, *P* < 0.01; ***, *P* < 0.001.

**FIG 8:**
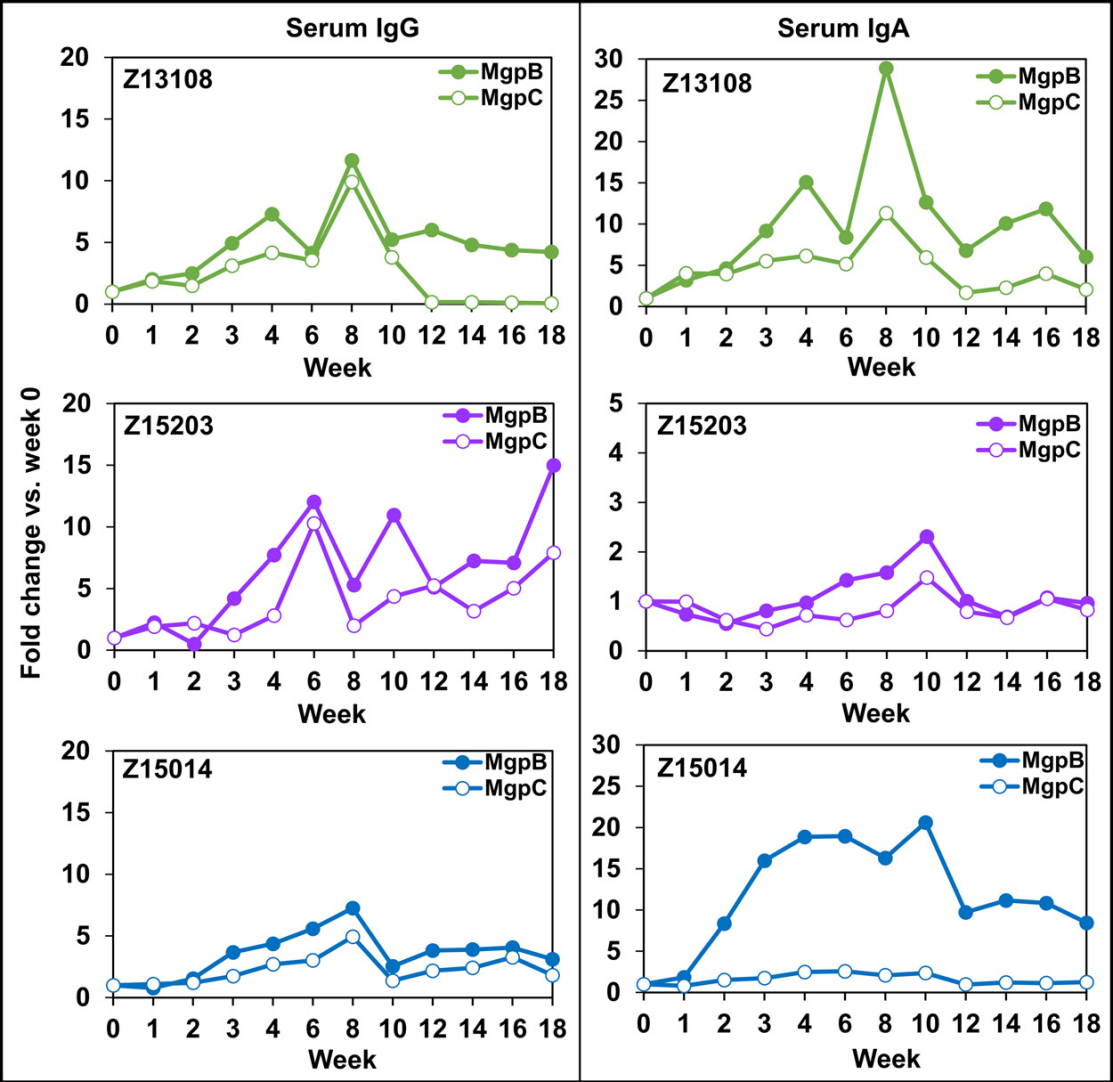
Serum reactivity to MgpB and MgpC over time. Primate numbers and week postinoculation are indicated. Fold change in IgG (left panels) and IgA (right panels) reactivity compared with week 0 are shown. IgM reactivity (not shown) was negligible in all three primates.

ELISAs detected increasing serum IgG reactivity to methanol-fixed whole M. genitalium cells in each primate (Fig. 7) and immunoblots determined that the MgpB and MgpC adhesins were the primary targets of serum antibodies (Fig. S2). Immunoblot band intensities were quantified and compared with pre-inoculation reactivity as shown in Fig. 8. IgG targeting MgpB and MgpC appeared after 3 weeks of infection and increased until approximately week 6 to 8. Subsequently, reactivity decreased somewhat in primates Z13108 and Z15014 but notably increased at week 18 in primate Z15203. IgA reactivity varied between primates: Z13108 reacted strongly with both MgpB and MgpC, Z15203 reacted minimally to both MgpB and MgpC, and Z15014 IgA reacted more robustly to MgpB than to MgpC (Fig. 8; Fig. S2). M. genitalium-reactive serum IgM did not increase compared with week 0.

M. genitalium antibody reactivity was detected at the site of infection in cervical swab specimens (Fig. 2; Fig. S2). Similar to serum antibodies, cervical antibodies reacted primarily with the MgpB and MgpC adhesin proteins and IgG was the dominant isotype, increasing more than 100-fold in all three primates. Increases in IgA and IgM reactivity to MgpB (but not MgpC) were detected in cervical secretions of Z13108 and Z15203, but not Z15104. Interestingly, peak cervical IgG reactivity coincided with a decrease in M. genitalium genomes in primates Z13108 and Z15203 (Fig. 2).

We considered whether antibody reactivity detected in cervical swabs could be attributed to the presence of menstrual blood. Using hemoccult assays, blood was detected in cervical specimens collected at weeks 2 and 16 for Z13108, weeks 3, 8, and 16 for Z15203, but in no specimens from Z15014. As the presence of blood did not correlate with immunoblot reactivity, we conclude that anti-M. genitalium IgG was produced locally or from transudation of serum into the genital tract.

### Biologic activity of primate serum.

**FIG 9:**
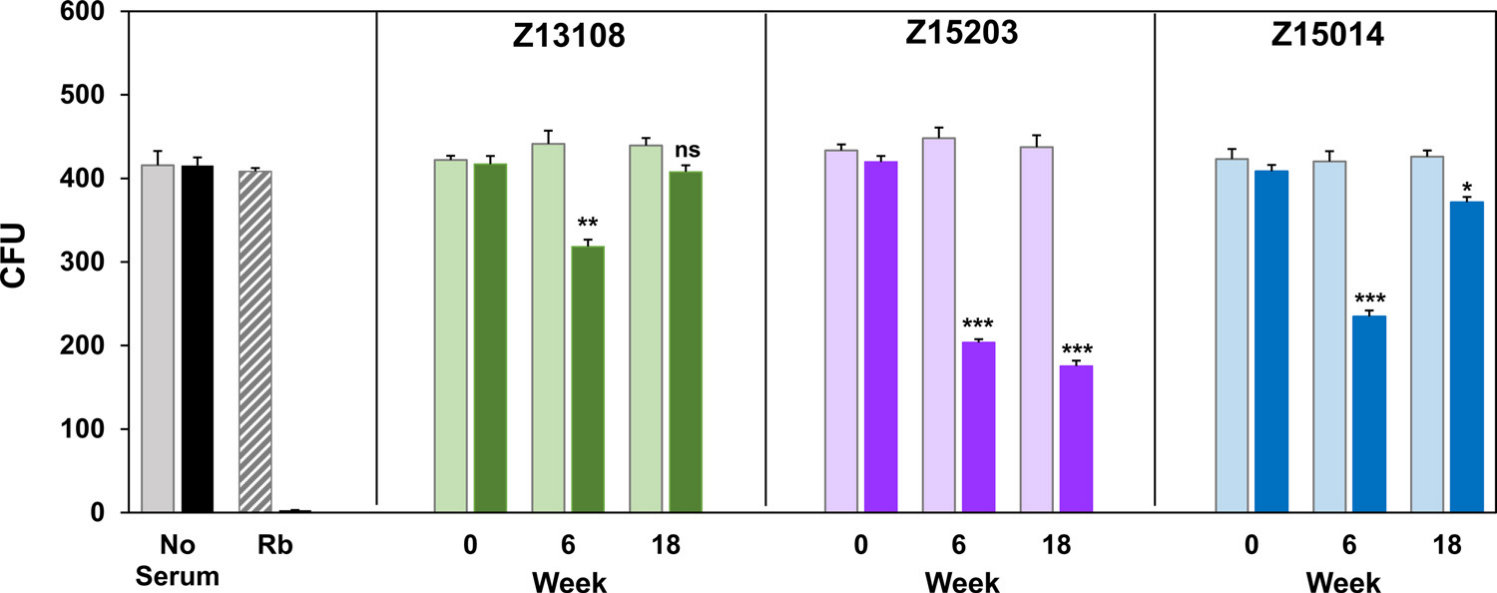
Complement-mediated killing of M. genitalium by primate sera. Average CFU (left) and serum treatment (bottom) are indicated. M. genitalium was treated with primate serum and active (dark colored bars) or heat-inactivated (light colored bars) human complement. Rb, rabbit anti-MgpB antibody positive control. Error bars indicate standard deviation of triplicate plate counts. Results of a typical experiment repeated three times are shown. *P* values were calculated using a two-tailed *t* test for means compared to week 0 serum; ns, *P* > 0.05; *, *P* < 0.01; **; *P* < 0.01; ***, *P* < 0.001.

We measured the biologic activity of primate antibodies *in vitro* using two assays: complement-mediated killing and metabolism inhibition. Complemented-mediated killing assays measure the bactericidal effect after a 1-h exposure to serum and complement. As shown in Fig. 9, sera collected at week 6 from all three primates induced complement-mediated killing. Interestingly, week 18 serum from primate Z13108 and Z15014 had reduced activity whereas serum from primate Z15203 maintained complement killing activity at week 18. Metabolism inhibition assays were used to measure growth inhibition in cultures continuously exposed to complement plus serial dilutions of serum from all time points. As shown in Table 2, Z13108 and Z15014 serum titers peaked at week 8 with a 4- and 16-fold increase in titer, respectively, and then declined thereafter. In contrast, Z15203 serum titers increased to 1,280 (week 16) and remained high at week 18 (Table 2). Control wells with complement only did not inhibit M. genitalium growth.

**TABLE 2:** Metabolism inhibition assay^*a*^

Week	Z13108	Z15203	Z15014
0	<10	<10	<10
1	<10	<10	<10
2	<10	<10	<10
3	<10	<10	20
4	20	40	80
6	10	160	80
8	40	320	160
10	40	320	80
12	10	640	40
14	10	640	40
16	<10	1280	20
18	<10	640	20

a Values indicate the maximum serum titer (reciprocal of dilution) at which growth inhibition was observed. The experiment was repeated three times with similar results.

## DISCUSSION

An animal model to study the natural history of M. genitalium infection, including mechanisms of persistence and role in upper reproductive tract disease, was recently identified as a prioritized research gap (2). We previously established that pig-tailed macaques are susceptible to M. genitalium infection, although not all animals were infected, lower genital tract persistence varied from 1 to ≥8 weeks, cervical infection induced antibodies in only a few animals, and invasion of the upper reproductive tract was not detected (16). Here, we report an optimized protocol with (i) improved infection rates, (ii) persistence for 16 to 18 weeks, (iii) induction of serum and cervical antibodies, (iv) inflammation of the lower genital tract, and (v) ascension to the upper reproductive tract in two animals.

M. genitalium was detected in lower genital tract specimens spanning 16 to 18 weeks in all three primates. In general, organism load was high (10^5^ to 10^6^ genomes per swab) in the first several weeks after inoculation, decreased at midpoint to near or below the PCR detection threshold (<10^3^ genomes per swab), then rebounded somewhat to an average of 10^4^ genomes per swab. Although we were unable to culture M. genitalium from later time points, we conclude that viable organisms were present because genome numbers increased, and previous experiments demonstrated that M. genitalium DNA is undetectable within 2 weeks of antibiotic treatment (15, 16). Organisms detected later in infection may derive from the upper reproductive tract as observed by Moller et al. (17) in which M. genitalium was cultured intermittently from the vagina after oviduct inoculation of female marmosets.

We detected M. genitalium in multiple upper reproductive tract tissues of primate Z13108 and Z15014 at week 18. In previous experiments (16) M. genitalium was not detected in the fallopian tubes after 4 or 8 weeks of infection, suggesting that more time is required for M. genitalium to reach this site. The lack of upper tract infection in Z15203 may be due to a more robust immune response as this primate produced more MIP1α and MIP1β chemokines, and serum from this primate was more effective at complement-mediated killing (see below). Interestingly, M. genitalium was detected in fallopian tubes but not in the endometrium of primate Z13108. This may suggest that (i) colonization of the endometrium is patchy and testing single endometrial biopsies may be inadequate to detect M. genitalium, and/or (ii) colonization of the uterus by M. genitalium is transient due to shedding of the endometrium during menses. In addition, although primate Z15014 had the highest number of M. genitalium genomes detected in the upper tract, only one lower genital tract specimen (endocervical) was PCR positive at the same time point. This suggests that women with M. genitalium-negative cervicovaginal specimens may harbor infection in the upper reproductive tract similar to N. gonorrhoeae or C. trachomatis (18).

M. genitalium infection was associated with intermittent increases in cervical PMNs and proinflammatory cytokines, and with histologic signs of cervicitis and vaginitis, but not with inflammation of the uterus or fallopian tubes, or with increased cervical exudate. It is not surprising that overt signs of cervical inflammation were absent given that the majority of M. genitalium infected women are asymptomatic (19), and that M. genitalium infection is more often associated with microscopic signs of cervicitis (i.e., infiltration of PMNs) than with clinical signs (i.e., cervical exudate) (20). Lewis et al. (21) estimated 4.9% of M. genitalium infections progress to pelvic inflammatory disease. In our model, M. genitalium frequently infects the fallopian tubes; however, salpingitis may develop in a subset of individuals, after longer infections, or after repeated infections as observed for C. trachomatis (22).

As we demonstrated, longitudinal analysis of the antibody response is valuable to discern immunologic patterns. Serum IgG specific to the MgpB and MgpC adhesin proteins appeared within 3 weeks and persisted throughout follow-up. Interestingly, higher complement killing activity was associated with the apparent clearance of M. genitalium in primate Z15203. In contrast, biologic activity of serum from Z13108 and Z15014 diminished late in the experiment and M. genitalium was not cleared. In comparison with serum antibodies, cervical MgpB/C-specific IgG reactivity peaked 4 to 8 weeks postinoculation then decreased thereafter. In two primates (Z13108 and Z15203), peak antibody reactivity coincided with declining organism load suggesting that antibodies were at least partially effective in clearing M. genitalium from the lower reproductive tract. In all three primates, M. genitalium genomes rebounded 10- to 100-fold after weeks 10 to 12 and persisted for an additional 6 to 8 weeks, when cervical antibodies were decreased, consistent with the hypothesis that M. genitalium can persist at low levels without stimulating, or perhaps suppressing (9), the local immune response. Of particular interest is whether the M. genitalium surviving the local antibody response consist of MgpB/C antigenic and phase variants that may escape antibody recognition (23).

Genital tract cytokines in M. genitalium-infected women have been reported in only a few studies. Wang et al. found that (i) vaginal cytokines varied widely in their study population, (ii) that there were no differences in cytokines when comparing women with and without M. genitalium infection, and (iii) women infected with both M. genitalium and C. trachomatis had lower cytokines than women infected with only C. trachomatis, suggesting that M. genitalium modulates the immune response (9). On the other hand, Garza et al. found that M. genitalium infection is associated with increases in some cytokines (VEGF-A, IL-1β, and IL-8) and decreases in others (IFN-γ, IL-12, IL-10) (24). Furthermore, increased cervical IL-1β, IL-6, and IL-8 were detected among HIV positive women with cervicitis (25). *In vitro* studies show that M. genitalium induces IL-6, IL-7, IL-8, and MCP-1 by cultured endocervical cells (26). In our primate model, we measured cytokines in endocervical secretions and found wide variations over time and between animals; however, IL-6 and IL-8 were increased in the majority of samples. The large differences in organism load, changes in sites of infection (lower versus upper genital tract), and differing stages of the menstrual cycle could have contributed to this variation. Taken together with the intermittent infiltration of PMNs our data suggests that M. genitalium induces low level inflammation punctuated by sporadic or localized increases in cytokine production. As M. genitalium grows as adherent microcolonies, and lipid associated membrane proteins activate cytokine secretion (27), we hypothesize that cytokines are produced by cells directly infected by M. genitalium. In this respect, cytokine staining of infected tissues may be more informative.

We conclude that this model is well suited to study factors important for colonization, persistence, clearance, and ascension to the upper reproductive tract. This model is translatable to human disease due to the similarity with humans including reproductive tract anatomy, menstrual cycle, and flora (28). As all infected animals produced antibodies, this model will be ideal to explore the role of antigenic and phase variation in persistence and immune evasion. Although our study is limited by the small number of animals, the correlation of antibody response with lack of histopathology or ascending infection suggests a properly targeted antibody response could be effective in limiting disease.

## MATERIALS AND METHODS

### Bacterial strains and growth conditions.

M. genitalium was routinely grown in SP-4 broth (29) containing 100 U/mL penicillin, at 37°C with 5% CO_2_. Primates were inoculated with M. genitalium strain G37-C, our single-colony filter-cloned isolate of the G37 type strain which has an *mgpB/mgpC* expression site and MgPar sequences identical to the fully sequenced genome (30).

We compared growth of M. genitalium strain G37-C in H broth and SP-4 broth as follows. M. genitalium (10^4^ genomes) was inoculated into 15 mL of each broth in 75-cm^2^ tissue culture flasks and cultured at 37°C with 5% CO_2_ until color change to orange indicated logarithmic growth (8 and 10 days for SP-4 and H broth, respectively). Bacteria were scraped into the culture supernatant, sheared 5 to 10 times through a 25G 1 1/2 inch needle to disrupt aggregates, then 10-fold serial dilutions were prepared in each broth, aliquots of which were spotted onto SP-4 agar plates. Broth dilutions and plates were incubated for 3 weeks to determine color change units and CFU per mL. Quantitative PCR (see below) was used to determine genomes per mL.

Using SP-4-grown M. genitalium we explored methods to prepare the inoculum. First, seed stocks were produced from a log phase culture and frozen in multiple 0.5 mL aliquots at −80°C. To prepare the inoculum, a 25-cm^2^ tissue culture flask containing 5 mL of SP-4 was inoculated with 0.5 mL of seed stock and cultured for 2 days at 37°C/5% CO_2_. Adherent bacteria were scraped into the culture supernatant then divided between two 75-cm^2^ tissue culture flasks each containing 15 mL SP-4 and incubated for an additional 2 days. Culture supernatants were discarded and adherent M. genitalium were washed twice with 5 mL of either phosphate-buffered saline (PBS) or sucrose phosphate glutamate (SPG) buffer, scraped into 1 mL, then sheared six times through a 25G 1 1/2 inch needle to disrupt clumps. Serial 10-fold dilutions were prepared in SP-4 broth, aliquots of which were spotted onto SP-4 agar plates. Broth and plates were incubated for 3 weeks to determine color change units and CFU per mL. We performed three trials comparing PBS to SPG buffer and found that PBS and SPG were equivalent for producing a homogeneous inoculum as measured by CFU, broth dilution (to determine color change units, CCU/mL), and genomes per mL. However, PBS was superior in maintaining 100% viability after storage for 3 h at room temperature (to simulate holding/transportation time before inoculation).

### Ethics statement.

Prior approval was obtained from the Institutional Animal Care and Use Committee (IACUC) at the University of Washington and from the Washington National Primate Research Center where the primates were housed. In this pilot study, three sexually mature female pig-tailed macaques (Macaca nemestrina) ages 5 to 7 years of age weighing 5.3 to 9.3 kg were enrolled. Animals were housed separately throughout the protocol with veterinary oversight and monitored closely for signs of distress. At study endpoint, animals were humanely euthanized in compliance with American Veterinary Medical Association Guidelines on Euthanasia and approved by IACUC.

### Reproductive tract infection model.

Vaginal, rectal, and cervical specimens were collected prior to, during, and after inoculation to confirm the absence of M. genitalium via qPCR, broth culture, and Vero cocultures (Table 1) and to confirm inhibition of normal flora by our antibiotic cocktail (penicillin at 100 units/mL, and colistin, vancomycin, and cefotaxime each at 50 μg/mL). At each time point, specimens were collected as follows: 10 mL blood was collected with silicone-coated Vacutainer blood collection tubes (Becton, Dickinson and Company, Franklin Lakes, NJ), serum was separated by brief centrifugation, and frozen at −80°C until analysis. Floqswabs (Copan Diagnostics, Murrieta, CA) were rolled on the vaginal epithelium and suspended in 2 mL mycoplasma transport medium (MTM) (31). A second vaginal swab specimen was suspended in 2 mL PBS for antibody analysis. Cervical exudates were collected with Merocel sponges (Beaver-Visitec Waltham, MA) applied to the cervical os for 2 min and stored dry at −80°C for cytokine detection. Cervical swabs were rolled on the face of the cervix; the first swab was suspended in MTM, and the second swab rolled onto a glass microscope slide (for microscopy, see below) then suspended in PBS. Similarly, two endocervical specimens were collected by inserting and rotating a cervical cytobrush (CooperSurgical, Trumbull, CT) in the endocervical canal. Cervical cytobrush samples were not collected at the time of inoculation (week 0) to avoid damage to the epithelium that may affect M. genitalium infectivity. Rectal swabs were collected at each time point for future studies. Rectal and cervical lavages (for future studies) were collected at weeks −3 and 18 by instilling 8 mL normal saline into the cavity and retrieving the sample with a syringe. At each time point the cervix was examined by colposcopy before and after specimen collection to identify gross changes in the appearance of the cervix.

To inoculate, we applied >2 × 10^9^ genome equivalents of freshly prepared M. genitalium in 1 mL PBS to the cervical os and ectocervix using a cannula. Primates Z13108 and Z15203 were inoculated with the same G37-C inoculum which contained 8.03 × 10^9^ genomes per mL, >1 × 10^8^ CCU per mL, and 3.2 × 10^8^ CFU per mL. The inoculum for Z15014 was similar: 3.8 × 10^9^ genomes per mL, >1 × 10^8^ CCU per mL, and 6.3 × 10^8^ CFU per mL. We confirmed there was no decrease in inoculum viability (CCU or CFU per mL) after inoculation (approximately 4 h later).

In addition to the specimens described above, specimens were collected at necropsy (week 18) from the upper reproductive tract including segments from the fallopian tubes (isthmus, ampulla, and fimbriae, ~0.5-1 cm in length), and the endometrium (~0.5 to 1 cm^2^). Tissue grinders (VWR, Radnor, PA) were used to homogenize tissue fragments in 2 mL MTM for M. genitalium qPCR and culture. Additional tissue samples were fixed for histology, stained with hematoxylin and eosin, and examined by a board-certified veterinary pathologist.

### Culture of M. genitalium from primate specimens.

Primate specimens in MTM or PBS were stored on ice until processing for culture within 4 h to 5 h. To maximize recovery of genital tract specimens, swab- and cytobrush-heads were snipped off and placed into an empty spin column within a collection tube. After centrifugation (2 min at 21,130 × *g*, 4°C), the eluted volume was combined with the original specimen. Viable M. genitalium were cultured from lower reproductive tract specimens in 35 mm petri plates containing 2 mL SP-4 broth supplemented with antibiotics to suppress growth of normal flora (penicillin at 100 U/mL, and colistin, vancomycin, and cefotaxime each at 50 μg/mL). SP-4 cultures were inoculated with 0.2 mL of specimen in MTM, then diluted through three additional 10-fold serial dilutions. Cultures were incubated at 37°C with 5% CO_2_ for up to 28 days; growth was detected by color change to orange or yellow (indicating glucose fermentation) and by microscopy to confirm the presence of adherent M. genitalium microcolonies.

In addition to SP-4 broth cultures, primate specimens were inoculated into Vero cocultures as previous experiments indicated this method was superior to H broth culture (16). In brief, 25-cm^2^ flasks were seeded with 1 × 10^5^ Vero cells in 5 mL Eagle’s Minimum Essential Medium (EMEM) supplemented with 10% fetal bovine serum (FBS) and 100 U/mL penicillin and then incubated overnight at 37°C in 5% CO_2_. The culture supernatant was discarded, adherent cells were washed with 5 mL PBS, and 8 mL of fresh EMEM supplemented with 10% FBS, 6% yeast dialysate, and antibiotics (100 U/mL penicillin, 50 μg/mL each of vancomycin, colistin, and cefotaxime) was added. After inoculation with 0.2 mL of primate specimen, cultures were incubated for 28 days collecting aliquots of culture supernatants every 7 days to detect an increase in M. genitalium genomes over time by qPCR. Control flasks inoculated with MTM alone were always negative for M. genitalium by qPCR.

### PCR detection of M. genitalium.

Total DNA was isolated from 150 μL of sample (inoculum, primate specimens, SP-4 broth, Vero coculture supernatants) using the MasterPure Complete DNA and RNA Purification kit (Lucigen, Middleton, WI) and suspended in 25 μL TE. M. genitalium genomes were quantified as previously described (16) using a modification of Jensen et al. (32). Each reaction contained 2 μL of extracted DNA (corresponding to 12 μL of specimen), 0.6 μM primers (MgPa-355F and MgPa432R), 125 nM 6-carboxyflourescein (FAM)-labeled probe (MgPa-380), and TaqMan Fast Universal PCR Mastermix No AmpErase UNG (Applied Biosystems, Waltham, MA) in a final volume of 10 μL. PCRs were amplified as follows: 95°C for 10 min, followed by 40 cycles of 95°C for 1 s and 60°C for 15 s on the Applied Biosystems QuantStudio & Flex real-time PCR system. Each sample was quantified in triplicate and compared to a standard curve prepared with serial 10-fold dilutions of M. genitalium DNA in quadruplicate. Specimens with ≥10 genome copies were considered PCR-positive based on comparison to pre-inoculation samples.

### Differential staining and microscopy of cervical smears.

Cervical smears collected throughout the observation period were differentially stained with Hemacolor Stain Set according to the manufacturer’s protocol (Sigma-Aldrich, Burlington, MA). PMNs were counted in five nonadjacent high-power fields (400x) and averaged.

### Detection of genital tract cytokines.

Endocervical secretions were extracted from Merocell sponges as described previously (33, 34). In brief, thawed sponges were equilibrated in 300 μL extraction buffer (PBS, 100 μg/mL aprotinin, 0.1% sodium azide, 0.25 M NaCl) for 30 min at 4°C and then centrifuged in a filter column at 13,520 × *g* for 20 min. The elution was repeated and the extracted volumes were combined. Cytokines and chemokines in eluted secretions were measured using the U-PLEX Pro-inflammatory and Chemokine kits for nonhuman primates (Meso Scale Discovery, Rockville, MA) and multiplied by the dilution factors. Dilution factors were determined as previously described (34): [(x – y) + 0.6g of buffer]/(x – y), where x is the weight of the sponge after absorbing cervical secretions and y is the mean weight of dry spears. Cytokines were measured in duplicate, and concentrations determined using Mesoscale Discovery Workbench analysis software (version 4.0). The light intensities from the samples were interpolated using a four-parameter logistic fit to a standard curve of electrochemiluminescence generated from known concentrations. The lower limit of detection for each marker can be found on the manufacturer’s website.

### Immunoblot assays.

Serum and cervical swab specimens suspended in PBS from each time point were collected and frozen at −80°C for later analysis. Immunoblots were used to measure antibody responses as reactivity to multiple M. genitalium antigens could be determined simultaneously, reactivity to MgpB and MgpC could be appreciated, and to take advantage of the wide dynamic range afforded by fluorescent antibody detection using the with Odyssey Clx LiCor infrared imaging system. M. genitalium G37-C lysates were prepared from bacteria grown in SP-4 broth. Lysates (2.5 μg total protein per lane, determined using the Pierce BCA Protein assay kit, Thermo Fisher, Waltham, MA) were electrophoresed through a 4% to 15% SDS-polyacrylamide gel (Bio-Rad, Hercules, CA) with unstained protein molecular weight standards (New England Biolabs, Ipswich, MA), transferred to a nitrocellulose membrane, and then reacted to primate serum (1:100), cervical swab specimen (1:50), or rabbit anti-MgpB and MgpC control sera (15). Bound IgG was detected using IRDye 800CW goat anti-human IgG or IRDye 680RD goat anti-rabbit IgG (Li-Cor, Lincoln, NE), diluted at 1:20,000, and imaged with Odyssey Clx LiCor infrared imaging system to quantify band intensity. Similarly, colorimetric detection of bound IgA and IgM were measured using peroxidase-conjugated goat anti-human secondary antibodies (Sigma-Aldrich, Burlington, MA), diluted at 1:2,000, and imaged with Bio-Rad ChemiDoc MP imaging system. Band intensities of MgpB- and MgpC-specific serum antibodies were quantified using the respective imaging system.

### Hemoccult assay.

Cervical specimens were tested for the presence of blood using the Sure-Vue fecal occult blood slide test system (Fisher Healthcare, Houston, TX). Cervical swabs suspended in PBS (5 μL) were applied to the slip, allowed to air dry, and then reacted with a drop of the kit developer solution. Traces of blood were identified by the production of a blue color. Results were recorded within 2 min of developing.

### Complement-mediated killing assay.

M. genitalium strain G37C was grown to mid-log phase in a 35 mm petri plate with 2 mL SP-4. Adherent bacteria were washed with Hanks’ balanced salt solution (HBSS, with Ca^2+^/Mg^2+^, no phenol red), scraped into 1 mL SP-4, and passed through a 0.45 μm filter. In a 0.5 mL tube, reactions were prepared with 30 μL of HBSS, 12.5 μL of the inoculum, 5 μL of heat-inactivated primate serum, and 2.5 μL IgG/IgM-depleted human complement (Pel-Freez Biologicals, Rogers, AR). Heat-inactivated serum was diluted 1:2,000 (Z13108) or 1:500 (Z15203 and Z15014) corresponding to the dilution at which week 0 serum did not affect viability. Heat-inactivated rabbit anti-MgpB serum diluted 1:20 (23) was used as a positive control. Each reaction was incubated at 37°C for 1 h, serially diluted, then spotted in triplicate onto SP-4 agar plates. Colonies were counted by light microscopy after 10 days incubation.

### Metabolism inhibition assay.

Metabolism inhibition tests were adapted from Taylor-Robinson et al. (35). Briefly, adherent, log phase M. genitalium growing in 5 mL SP-4 broth were washed with HBSS, scraped into 3 mL of SP-4, passed through a 0.45 μm filter, and diluted 1:20 in SP-4. Two-fold serial dilutions of heat-inactivated serum were prepared in a 96-well plate containing 100 μL of SP-4 per well. Guinea pig complement (20 μL of 1:4 dilution; MP Biomedicals, Irvine, CA) and diluted inoculum (20 μL) were added for a total volume of 200 μL per well. Plates were incubated at 37°C with 5% CO_2_ for 8 days. The highest dilution at which antiserum inhibited M. genitalium growth as determined by color change was considered the endpoint.
