# Supplementary material for: Ascending Reproductive Tract Infection in Pig-Tailed Macaques Inoculated with Mycoplasma genitalium
Source: Infect Immun. 2022 May 18;90(6):e00131-22. doi: 10.1128/iai.00131-22 (PMC9202418; doi:10.1128/iai.00131-22)
Supplement: Supplemental file 1 — Fig. S1 and S2. Download iai.00131-22-s0001.pdf, PDF file, 4.1 MB [file iai.00131-22-s0001.pdf]

Supplemental Figure 1A. Endocervical cytokines detected in *M. genitalium*-infected primates.

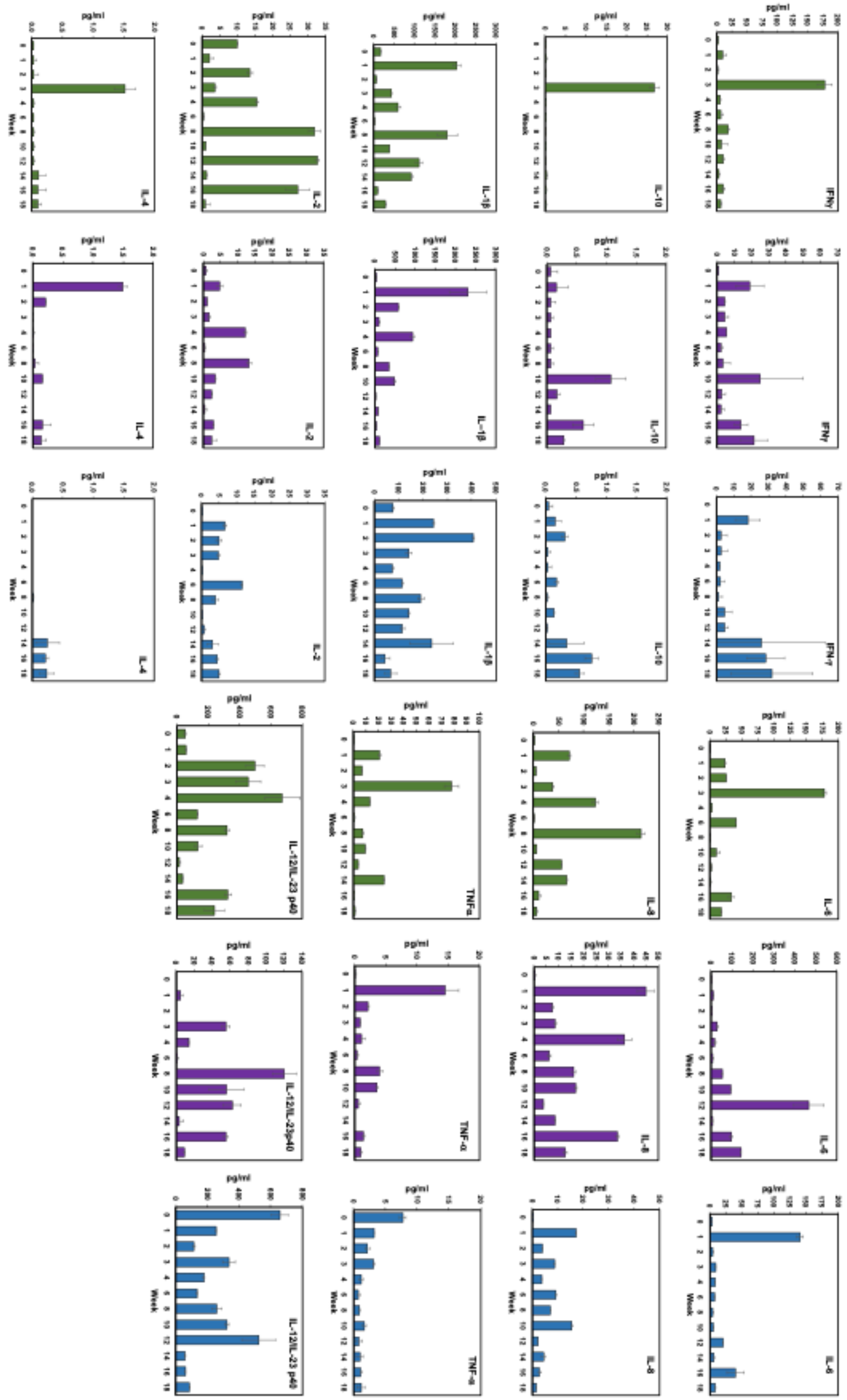

Supplemental Figure 1B. Endocervical chemokines detected in M. *genitalium*-infected primates.

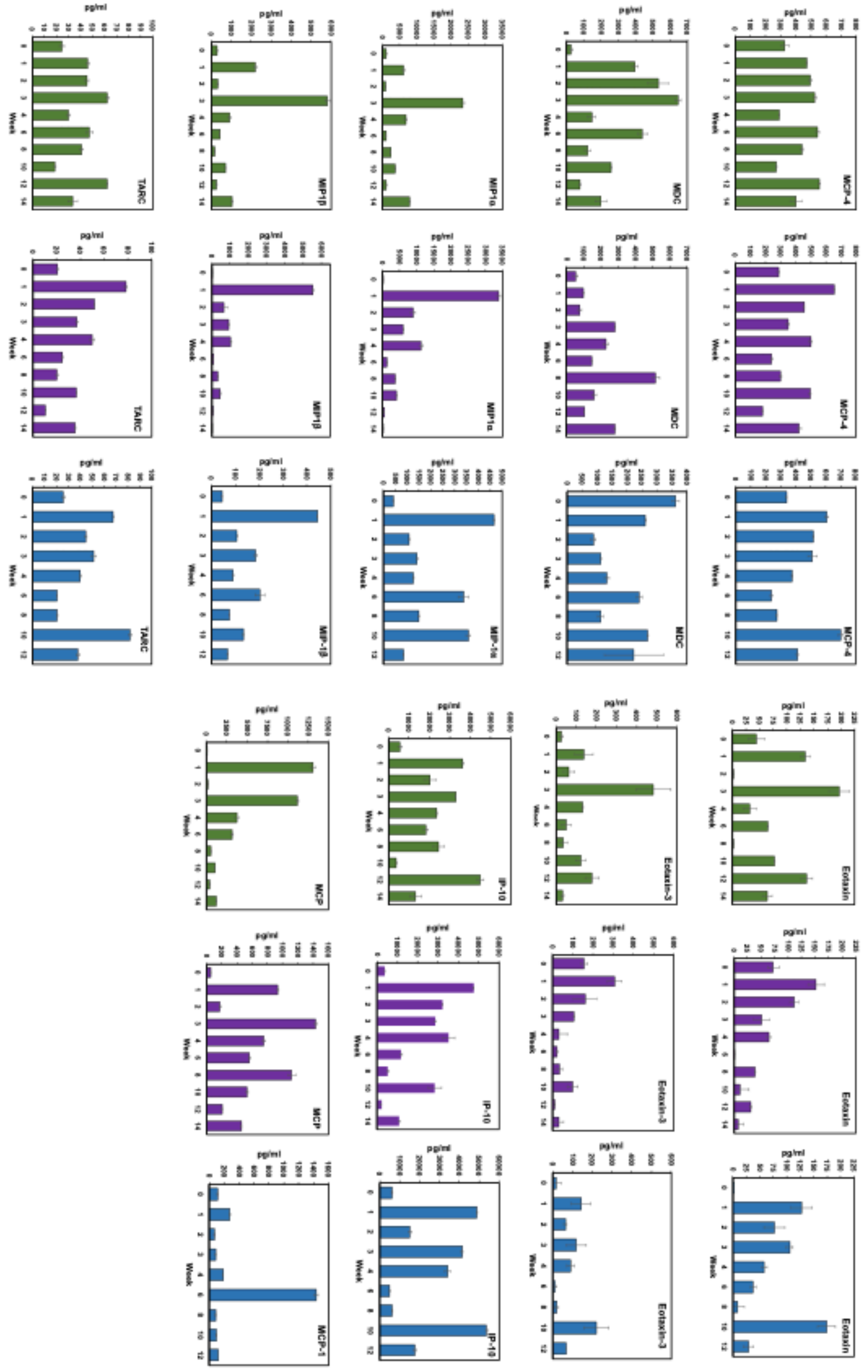

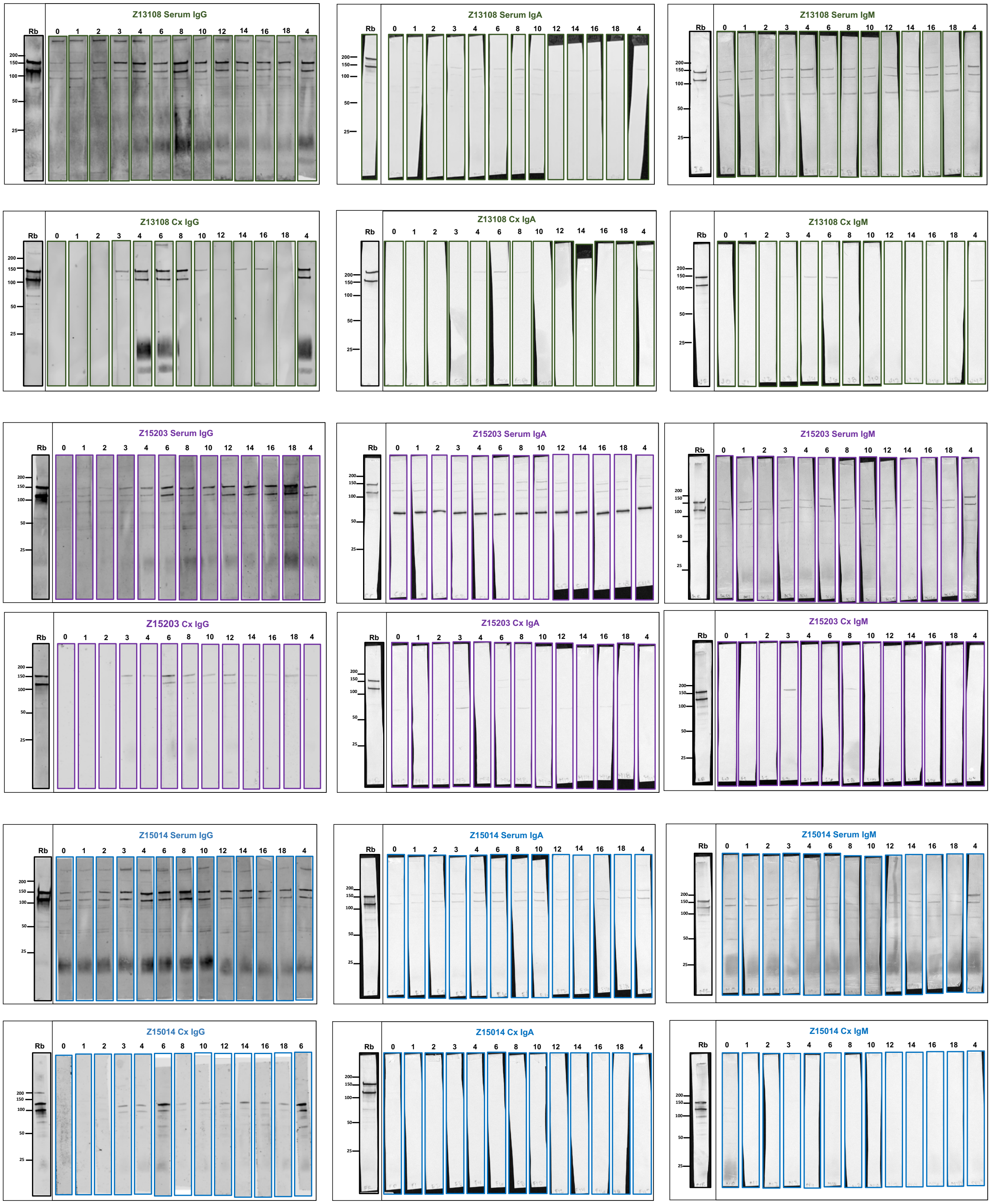

Supplemental Fig 2 Immunoblot reactivity of primate serum and cervical (Cx) swab specimens with *M. genitalium* whole cell lysates. Rb, rabbit anti-MgpB and anti-MgpC antibody reactivity. Numbers above strips indicate week after inoculation. As two gels were needed to span all time points, week 4 serum or cervical specimen was included on both gels to normalize reactivity. Molecular weight markers are indicated at left.
